# Survival Patterns in United States (US) Medicare Enrollees with Non-CML Myeloproliferative Neoplasms (MPN)

**DOI:** 10.1371/journal.pone.0090299

**Published:** 2014-03-11

**Authors:** Gregory L. Price, Keith L. Davis, Sudeep Karve, Gerhardt Pohl, Richard A. Walgren

**Affiliations:** 1 Eli Lilly and Company, Indianapolis, Indiana, United States of America; 2 RTI Health Solutions, Research Triangle Park, North Carolina, United States of America; Case Western Reserve University, United States of America

## Abstract

**Purpose:**

Non-CML myeloproliferative neoplasms (MPN) include essential thrombocythemia (ET), polycythemia vera (PV), and myelofibrosis (MF). Reported median overall survival (OS) ranges from a few to several years for MF, a decade or more for ET and PV. The study objective was to compare US survival rates of ET, PV, and MF patients with matched non-MPN/non-cancer controls in a nationally representative database.

**Patients and Methods:**

Data were taken retrospectively from the Survey, Epidemiology, and End Results (SEER)-Medicare linked database. Medicare enrollees with a new SEER MPN diagnosis between Jan 1, 2001 and Dec 31, 2007 were eligible. First MPN diagnosis was required at or after Medicare enrollment to allow for continuous follow-up. Non-MPN/non-cancer control groups were selected from Medicare separately for each MPN subtype and demographically matched to cases at a ratio of 5∶1. Survival was determined starting from the case diagnosis date using the Kaplan-Meier method.

**Results:**

A total of 3,364 MPN patients (n = 1,217 ET; 1,625 PV; 522 MF) met the inclusion criteria and were matched to controls. Mean age was 78.4, 76.1, and 77.4 years for ET, PV, and MF, respectively, and percent female was 63, 50, and 41. Median OS was significantly (p<0.05) lower for MPN cases vs. controls (ET: 68 vs. 101 months; PV: 65 vs. 104; MF: 24 vs. 106).

**Conclusions:**

In the US Medicare population, survival in MF patients was worse than that of patients with ET or PV and significantly worse than matched controls. Survival of patients with ET or PV was substantially inferior to matched controls. These findings have implications for the clinical management of MPN patients and underscore the need for effective therapies in all MPN subtypes.

## Introduction

Myeloproliferative neoplasms (MPNs) are a group of hematologic malignancies characterized by the clonal or oligoclonal proliferation of one or more myeloid lineages that arise from a polyclonal stem cell pool. The World Health Organization classification of MPNs [1] includes chronic myelogenous leukemia, polycythemia vera (PV), essential thrombocythemia (ET), myelofibrosis (MF), chronic neutrophilic leukemia, chronic eosinophilic leukemia not otherwise specified, mastocytosis, and MPNs not otherwise specified (MPN-NOS). The non-chronic myeloid leukemia (CML) MPNs ET, PV, and MF are characterized by activation of JAK2 signaling and abnormal blood cell production [2]–[4]. MPNs are rare with an incidence ranging from approximately 0.5 to 3 per 100,000 persons depending on the subtype and geography [5]–[8].

The non-CML MPNs are distinguished from one another on the basis of effects on cell lineages and the involvement of fibrosis in the bone marrow compartment [1]. PV is characterized by an increase in red blood cell production, or red cell mass (RCM), occurring independently of normal regulatory mechanisms. In PV, increases in the RBC mass may occur in concert with an increase in platelet numbers as well. Major diagnostic criteria for PV include increases in hemoglobin (>18.5 g/dl in males, >16.5 g/dL in females; or other evidence of increased red cell volume) and presence of JAK2 V617F or similar mutation [9]. While not included in the current WHO diagnostic criteria, an elevated RCM may assist in identifying PV patients who do not meet the defined elevations in hemoglobin or hematocrit values [10]. In contrast, ET involves overproduction of platelets in the absence of an increased red blood cell mass [11], [12]. MF is characterized by a progressive evolution or worsening of bone marrow fibrosis and abnormal blood cell production, with many patients initially demonstrating hypercellularity, but changes in hematopoiesis can lead to the development of a hypocellular state later in the evolution of the disease [13].

Survival rates of non-CML MPN patients, by subtype, from a nation-wide US population have not been recently described. MPNs are more prevalent in the elderly, and therefore Medicare enrollees are a highly relevant source for US-based survival estimates in these diseases. To address this knowledge gap, this study compared survival rates of Medicare enrollees diagnosed with MPNs (ET, PV, and MF) with matched non-MPN/non-cancer controls.

## Patients and Methods

### Data Source

Retrospective data were taken from the Survey, Epidemiology, and End Results (SEER)-Medicare linked database in the US, which combines clinical information from the SEER cancer registry (MPN reporting has been required since 2001) with medical and pharmacy claims for Medicare Part A and B enrollees. SEER areas have been shown to be nationally representative [14] and capture approximately one-quarter of the total US population [15]. For each incident cancer diagnosis reported in SEER, patient-level information is captured on demographics, date of diagnosis, clinical data about the malignancy (e.g., histology, morphology, topography, stage, grade), specific International Classification of Diseases for Oncology, Third Revision (ICD-O-3) codes, and survival. Medicare claims are currently linked through 2009 for Medicare-enrolled SEER patients with an incident cancer diagnosis between 1991 and 2007.

The research presented in this report was conducted with Institutional Review Board (IRB) approval in accordance with the Helsinki Declaration on the protection of human subjects. The research organization conducting this study, RTI Health Solutions, a business unit of RTI International, holds a Federal-Wide Assurance (FWA) from the US Department of Health and Human Services (DHHS) Office for Human Research Protections (OHRP) that allows for the review and approval of human subjects protocols through internal IRB committees. The ethics committee, Research Triangle Institute Committee for the Protection of Human Subjects (FWA #3331), reviewed this study to ensure adherence to appropriate regulations that govern human subjects research, including 45 CFR 46, 21 CFR 50 and 56, and all applicable International Conference on Harmonization provisions, including the Helsinki Declaration.

Because no new data were collected on human subjects (i.e., all data were retrospective, de-identified, and non-interventional), the study was exempted from patient consent requirements and was approved for conduct by the authorized IRB. Furthermore, this research followed rules of the SEER-Medicare Data Use Agreement (DUA) for external investigators that require suppression or combining of results fields comprising fewer than 11 patients in order to further ensure patient anonymity.

### Patient Cohort

Medicare enrollees with a new SEER MPN diagnosis between January 1, 2001 and December 31, 2007 were selected and followed on survival in the linked Medicare claims from first MPN diagnosis date (as reported in SEER) until death or end of follow-up in the linked claims data (December 31, 2009). Patients were classified by MPN subtype based on the ICD-O-3 code recorded in SEER (9962/3 for ET, 9950/3 for PV, 9961/3 for MF). First MPN diagnosis date was required to occur on or after first Medicare enrollment to allow for continuous follow-up until death or censoring at the end of the database. Thus, patients with an MPN diagnosis prior to their Medicare enrollment date were excluded. Non-MPN/non-cancer control groups were selected from the national 5% Medicare sample for each MPN subtype and matched to cases 5∶1 based on year of birth, gender, race, geographic location, and reason for Medicare eligibility (age or disability). Patients were excluded if reason for Medicare eligibility was end-stage renal disease (Figure S1).

### Survival Analyses

Survival for the matching controls was assessed from the diagnosis date of their respective match until death or until censoring at the end of the database. Survival was descriptively estimated and compared for MPN cases and controls using the Kaplan-Meier method. Separate Cox proportional hazards models were fit to each of the three diseased cohorts to investigate the prognostic impact of age group, gender, race and reason for Medicare eligibility on survival. Finally, using life table methods, the proportion of patients in each MPN subtype surviving at least 1, 3, 5, and 7 years after diagnosis was estimated and reported. All survival analyses were conducted in SAS (Version 9.3, Cary, NC) statistical software using the LIFETEST procedure.

## Results

A total of 3,364 MPN patients (n = 1,217 ET, 1,625 PV, 522 MF) were identified for inclusion and assigned matching controls (Table 1). Mean [SD] age was 78.4 [8.2], 76.1 [10.5], and 77.4 [7.9] years for ET, PV, and MF, respectively, while percent female was 62.5, 50.3, and 41.4. Disability was the Medicare eligibility reason for 10% of ET, 18% of PV, and 12% of MF patients. Non-white ethnicity was 13% of ET, 11% of PV, and 10% of MF patients.

**Table 1:** Patient Characteristics, by MPN Subtype.

	ET (n = 1,217)		PV (n = 1,625)		MF (n = 522)	
	N	%	N	%	N	%
**Age at MPN Diagnosis**						
≤64 years	62	5.1	184	11.3	22	4.2
65–74 years	305	25.1	442	27.2	163	31.2
75–84 years	608	50.0	730	44.9	271	51.9
≥85 years	242	19.9	269	16.6	66	12.6
Mean [SD] Age	78.4 [8.2]		76.1 [10.5]		77.4 [7.9]	
**Gender**						
Male	457	37.6	808	49.7	306	58.6
Female	760	62.5	817	50.3	216	41.4
**Race**						
White	1059	87.0	1450	89.2	470	90.0
Black	96	7.9	92	5.7	31	5.9
Other*	19	1.6	23	1.4	21	4.0
Asian	29	2.4	42	2.6	—	—
Hispanic	14	1.2	18	1.1	—	—
**Reason for Medicare Eligibility**						
Age	1095	90.0	1326	81.6	459	87.9
Disability	122	10.0	299	18.4	63	12.1

* Per SEER-Medicare privacy rules, cell sizes <11 have been supressed, requiring collapsed reporting of “Other” race as follows: ET: “Other” includes North American Native and Other race/ethnicity; PV: “Other” includes North American Native and Other race/ethnicity; MF: “Other” includes Asian, Hispanic, North American Native, and Other race/ethnicity.

**Figure 1:**
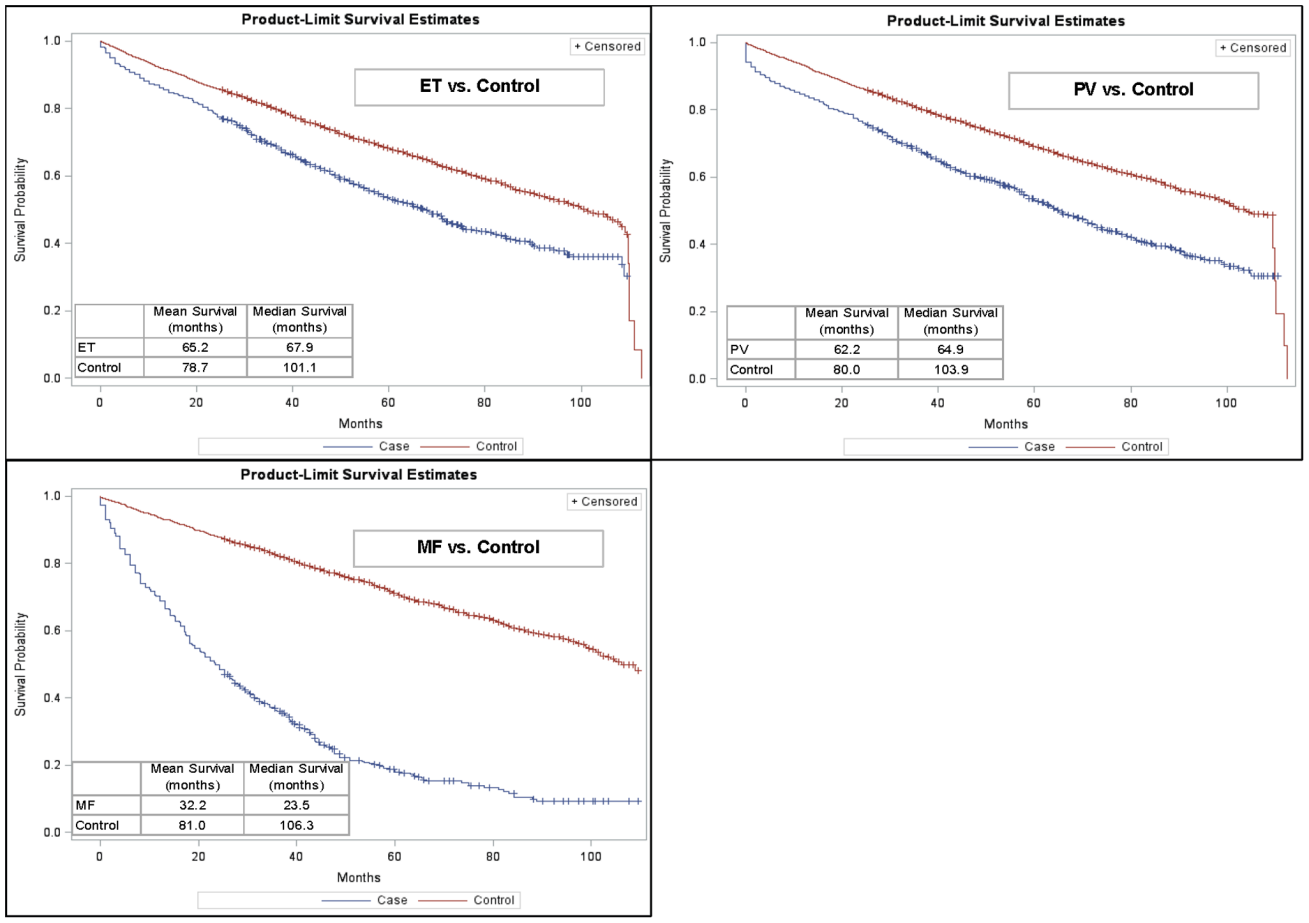
Kaplan-Meier Survival Estimates, by MPN Subtype. ET = essential thrombocythemia, PV = polycythemia vera, MF = myelofibrosis.

**Figure 2:**
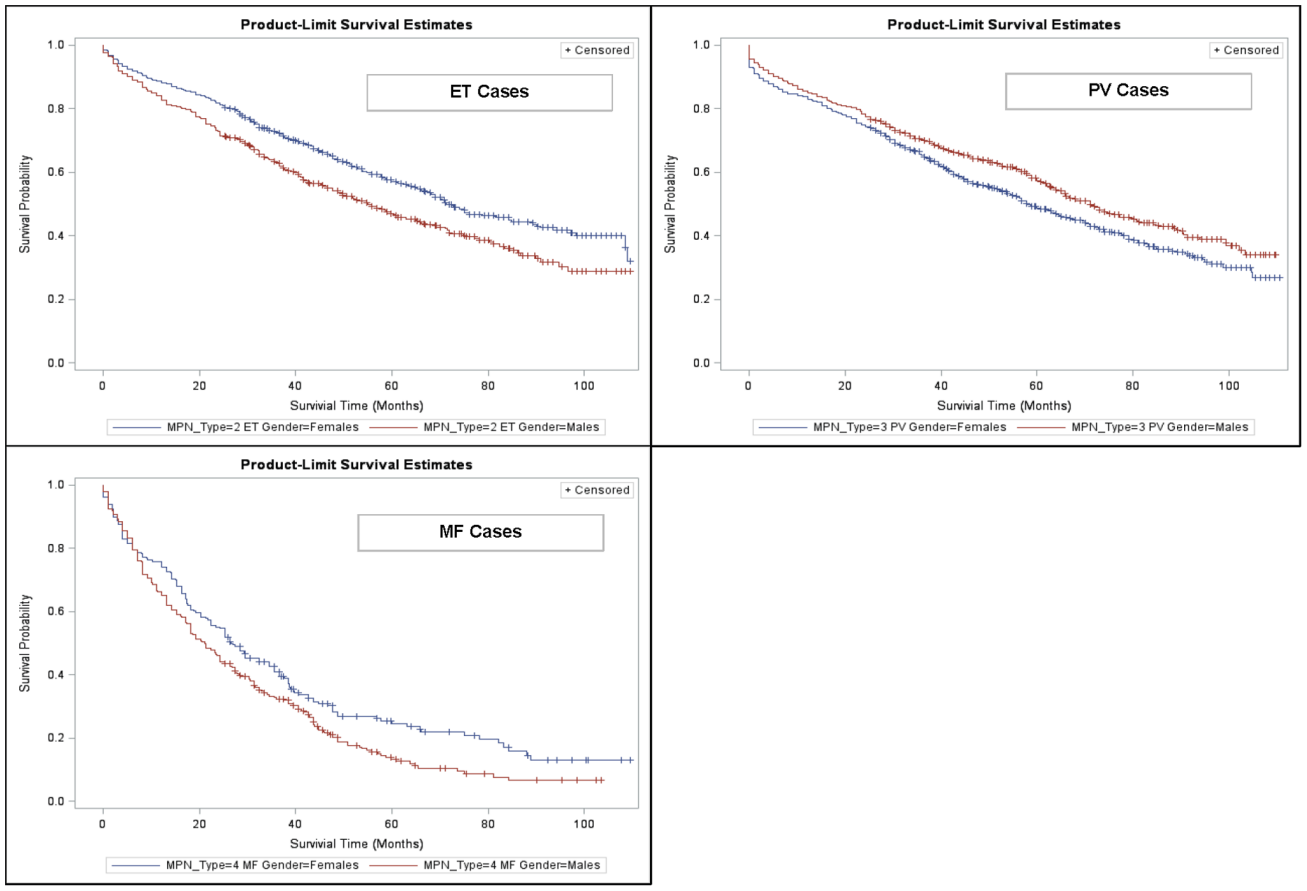
Kaplan-Meier Survival Estimates, by MPN Subtype and Gender. ET = essential thrombocythemia, PV = polycythemia vera, MF = myelofibrosis.

**Figure 3:**
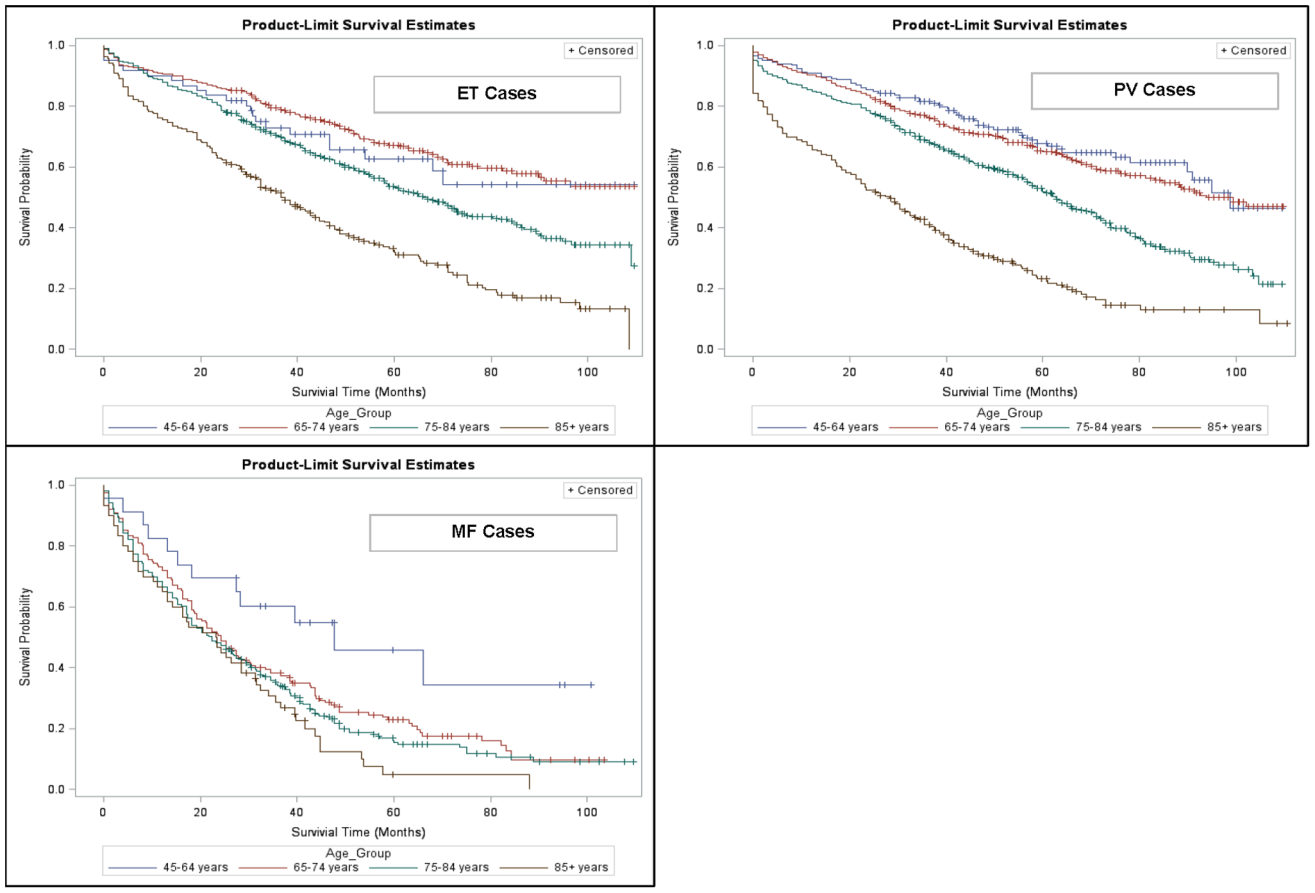
Kaplan-Meier Survival Estimates, by MPN Subtype and Age Group. ET = essential thrombocythemia, PV = polycythemia vera, MF = myelofibrosis.

Median overall survival was significantly (p<0.05) lower for MPN cases vs. controls (ET: 68 vs. 101 months; PV: 65 vs. 104 months; MF: 24 vs. 106 months) (Figure 1). These results were also reflected in the 1-, 3-, 5- and 7-year survival rates presented in Table 2. The 7-year survival rate for MF patients, for example, was only 11% compared with 42% for patients with ET. Females with ET and MF appeared to have better survival than males with these MPN subtypes (Figure 2). Females with PV, however, were found to have a survival disadvantage relative to males with PV. Increasing age was generally found to be inversely associated with survival, except for patients with ET in whom younger patients (age 45–64 years) had worse survival than patients 65–74 years of age (Figure 3). Various factors, including age group, gender, region and race were significant in the Cox proportional hazard models with the most salient by far being advanced age (Table 3).

**Table 2:** 1-, 3-, 5- and 7-Year Survival Rates Post-Diagnosis, by MPN Subtype.

Survival Time	ET (n = 1,217)	PV (n = 1,625)	MF (n = 522)
**1 year**	86.9%	84.6%	70.3%
**3 years**	69.1%	67.9%	36.0%
**5 years**	53.2%	53.1%	18.2%
**7 years**	42.0%	40.2%	10.7%

ET = essential thrombocythemia, PV = polycythemia vera, MF = myelofibrosis,

**Table 3:** Cox Proportional Hazard Regression Results by MPN Type.

Table 3a: Cox Proportional Hazard Regression Results: MPN type - ET				
	Hazard ratio	95% CI		P Value
**Age Groups (Ref: 45–64 years)**				
65–74 years	1.00	0.57	1.73	0.986
75–84 years	1.68	0.97	2.90	0.065
≥85 years	3.21	1.85	5.57	<.0001
**Gender (Ref: Females)**				
Males	1.43	1.21	1.68	<.0001
**Race (Ref: White)**				
Black	1.63	1.23	2.15	0.001
Other	0.88	0.59	1.32	0.545
**Region (Ref: West)**				
Northeast	1.13	0.90	1.40	0.291
Midwest	0.91	0.73	1.13	0.389
South	1.16	0.91	1.47	0.248
**Reason for Medicare Eligibility (Ref: Normal enrollment)**				
Disability	1.14	0.80	1.62	0.467

Table 3b: Cox Proportional Hazard Regression Results: MPN type - PV				
	Hazard ratio	95% CI		P Value
**Age Groups (Ref: 45–64 years)**				
65–74 years	1.36	0.94	1.97	0.099
75–84 years	2.26	1.55	3.29	<.0001
≥85 years	4.96	3.34	7.36	<.0001
**Gender (Ref: Females)**				
Males	1.00	0.87	1.15	0.983
**Race (Ref: White)**				
Black	1.51	1.14	1.99	0.004
Other	0.80	0.58	1.12	0.202
**Region (Ref: West)**				
Northeast	0.82	0.68	0.99	0.041
Midwest	0.90	0.74	1.09	0.273
South	0.82	0.67	0.99	0.038
**Reason for Medicare Eligibility (Ref: Normal enrollment)**				
Disability	1.20	0.91	1.57	0.196

Table 3c: Cox Proportional Hazard Regression Results: MPN type - MF				
	Hazard ratio	95% CI		P Value
**Age Groups (Ref: 45–64 years)**				
65–74 years	2.60	1.34	5.06	0.005
75–84 years	3.06	1.57	5.98	0.001
≥85 years	4.01	1.96	8.21	0.000
**Gender (Ref: Females)**				
Males	1.25	1.03	1.53	0.028
**Race (Ref: White)**				
Black	0.94	0.62	1.42	0.773
Other	0.74	0.44	1.27	0.275
**Region (Ref: West)**				
Northeast	1.16	0.88	1.52	0.293
Midwest	0.96	0.74	1.25	0.772
South	1.57	1.18	2.07	0.002
**Reason for Medicare Eligibility (Ref: Normal enrollment)**				
Disability	1.35	0.96	1.92	0.089

## Discussion

Table 4 briefly summarizes available studies on survival in MPN patients. Studies describing survival in US patients with MPNs are limited, and the few available feature small samples [16]–[18] or limited follow-up time [19]. Prior to 2012, only a limited number of observational studies on MPN survival were published from international populations. In Europe, country-specific studies have examined MPN survival in Spain [20], Italy [21], France [5], and Sweden [22]. A few pan-European studies also reported MPN survival [23], [24]. Additionally, one non-Caucasian report provided insight into the Chinese MPN population survival trends [25]. Lastly, potential reasons for survival disparity can also include limited geographic scope, referral bias, socio-economic differences, specificity for MPN (a single general category or by subtype), age and recency of data, all-comers in the US vs Medicare-only, treatment heterogeneity, inclusion of matched controls, study duration (one vs. several years) and oversampling for minorities.

**Table 4:** Summary of Available MPN Survival Estimates.

MPN Subtype	Author	Source	Population	Median OS (Years)	OS/RS/RSR
All MPN	D Rollison et al	Blood 2008; 112:45–52	3916 from US cancer registries	—	80% OS at 3 yrs
ET	M Hultcrantz et al	JCO 2012; 30(24):2995–3001	2559 pts from SW 1973–2008	—	.68 RSR at 10 yrs
ET	Maynadie M et al	Haematologica 2012; Sept 14 (epub)	1230 pts in 48 registries in 20 EU countries	—	89.9% RS at 5 yrs
ET	Maynadie M et al	Haematologica 2011;96(1):55–61	229 pts from Cote d'Or in France	—	60% OS at 10 yrs
ET	Mesa RA et al	Am J Hemat 1999;61:10–15	39 pts from Olmstead County, MN	10.8	—
ET	Passamonti F et al	Am J Med 2004;117:755–761	435 pts from two general hosp in IT	22.6	—
ET	S Malak S et al	Blood Cells Mol Dis 2012;49(3–4):170–6	105 pts from FR, BE 1998–2010	—	83% OS at 10 yrs
ET	Wolanskyj AP et al	Mayo Clin Proc 2006; 81(2):159–166	322 pts seen at Mayo Clinic in MN	18.9	—
PV	Ania BJ et al	Am J Hemat 1994;47(2):89–93	50 pts from Olmstead County, MN	7.2	—
PV	M Hultcrantz et al	JCO 2012; 30(24):2995–3001	4389 pts from SW 1973–2008	—	.64 RSR at 10 yrs
PV	Maynadie M et al	Haematologica 2012; Sept 14 (epub)	1382 pts in 48 registries in 20 EU countries	—	84.8% RS at 5 yrs
PV	Maynadie M et al	Haematologica 2011;96(1):55–61	116 pts from Cote d'Or in France	—	56% OS at 10 yrs
PV	Passamonti F et al	Am J Med 2004;117:755–761	396 pts from two general hosp in IT	20	—
PV	S Malak S et al	Blood Cells Mol Dis 2012;49(3–4):170–6	97 pts from FR, BE 1998–2010	—	83% OS at 10 yrs
MF	Cervantes F et al	JCO 2012;30(24):2981–7	434 pts from FR, IT, SP, UK	4.6	—
MF	Cervantes F et al	JCO 2012;30(24):2981–7	368 pts from FR, IT, SP, UK	6.5	—
MF	M Hultcrantz et al	JCO 2012; 30(24):2995–3001	1048 pts from SW 1973–2008	—	.21 RSR at 10 yrs
MF	S Malak S et al	Blood Cells Mol Dis 2012;49(3–4):170–6	14 pts from FR, BE 1998–2010	—	46% OS at 10 yrs
MF	Xu Z et al	Blood 2012; 119(11):2469–73	642 pts from a single hospital in China	6.6	—
MF	Mesa RA et al	Am J Hemat 1999;61:10–15	21 pts from Olmstead County, MN	3	—
MF	Maynadie M et al	Haematologica 2012; Sept 14 (epub)	249 pts in 48 registries in 20 EU countries	—	34.6% RS at 5 yrs
MF	Maynadie M et al	Haematologica 2011;96(1):55–61	43 pts from Cote d'Or in France	—	21% OS at 10 yrs
MPN-NOS	Maynadie M et al	Haematologica 2012; Sept 14 (epub)	1311 pts in 48 registries in 20 EU countries	—	55.3% RS at 5 yrs
MPN-NOS	M Hultcrantz et al	JCO 2012; 30(24):2995–3001	1388 pts from SW 1973–2008	—	.49 RSR at 10 yrs
MPN-NOS	Maynadie M et al	Haematologica 2011;96(1):55–61	25 pts from Cote d'Or in France	—	25% OS at 10 yrs

ET = essential thrombocythemia, MF = myelofibrosis, MPN-NOS = myeloproliferative disorder not otherwise specified, OS = overall survival, pts = patients, PV = polycythemia vera, RSR = relative survival rate.

Across most of the available studies, patients with MF were consistently reported to have a substantially reduced life expectancy, while patients with PV or ET were generally observed to have a good prognosis with only a slight reduction or no change in expected survival. In one of the studies, however, survival in ET patients was observed to be similar to controls only in the first decade after diagnosis and then significantly worsened thereafter [18]. A recent study in Sweden reported substantially reduced survival in all MPN subtypes over four calendar periods compared to the general population, while noting that survival improved in PV and ET patients after 1993 [22].

In our large population-based study including more than 3,300 Medicare enrollees with MPNs diagnosed between 2001 and 2007, survival in MF patients was significantly worse than that of patients with ET or PV. This finding is consistent with the previously summarized studies comparing survival in MPN patients versus non-MPN patients. Also consistent with findings by Hulcrantz et al. (2012) in a Swedish population [22], and contrary to previous reports that ET and PV patient's experience near-normal life expectancy, survival of patients with ET or PV was substantially inferior to matched controls in the Medicare population examined here.

Previous studies suggest that survival in MPN patients can be influenced by several factors. Consistent with many other cancer types, increased age is associated with decreased survival in MPNs [19], [22]–[24], while female gender is associated with improved survival [22]. MPN subtype influences survival, with PV and ET generally showing significantly longer survival that MF [22], [26]. *JAK2* V617F mutation status may also impact survival [27], but this factor is rarely examined or captured outside of clinical trial settings, particularly for PV and ET patients. Geography and ethnicity can also impact survival. Maynadie et al. (2012) reported regional survival differences between Northern Europe and Eastern Europe of 74% and 27%, respectively [23]. Xu et al. (2012) compared MPN patients in China with literature reports based primarily on Caucasian populations, finding that Chinese MPN patients were significantly younger, with fewer having palpable spleens or constitutional symptoms, and had significantly better survival as compared with Caucasian MPN patients [25].

Our survival findings based on age group were consistent with these prior reports. Interestingly, ET and PV patients 85 years of age or older in our study were well-separated from the younger three cohorts in terms of lower survival. However, in the MF group, the youngest cohort (45–64) separated with far better survival from the older three age groups, which had relatively similar survival. Improved survival based on female gender was mostly consistent with prior MPN research, with one exception for PV males, who had better survival than PV females. Compared to white patients with PV and ET, black patients in both subtypes experienced a statistically significant higher risk of death, possibly related to health care access challenges for patients with longer term MPN disease. Across the three MPN subtypes, approximately half of the MPN patients were eligible for the JAK2 V617F testing based on their diagnosis date, but very few results for this test were recorded in SEER. The reasons for this lack of data are unclear and preclude further survival analysis based on JAK2 V617F status. Disease associated risk factors such as age greater than 65 years, hemoglobin level, white blood cell count, peripheral blood blasts, constitutional symptoms, and abnormal karyotypes have been shown to have predictive value for assessment of survival using the Dynamic International Prognostic Scoring System for Primary Myelofibrosis [28], [29]. Except for age, the information was not available so that analysis based on risk was not conducted.

Our study was subject to several limitations. First, our study was limited to enrollees in the US Medicare system, which consists primarily of older patients. Thus, our findings may not be generalizable to other populations, including those enrolled in commercial managed care plans or patients in other public payer systems such as Medicaid. Second, although MPN incidence is highest in elderly persons, our Medicare sample was not representative of the true age distribution of all MPN cases in the US, and was specifically subject to underrepresentation of younger patients. Third, MPN patients in our study were required to have been first diagnosed with an MPN on or after Medicare entry. This inclusion criterion ensured that all patients could be followed continuously until death or censoring, but it resulted in the exclusion of some patients diagnosed with an MPN at younger ages who survived until Medicare entry. Our study sample may therefore be older than the general Medicare population living with these diseases. Lastly, while the SEER cancer registry is considered an authoritative source and system for the reporting of both solid tumor and hematologic malignancies in the US, recent evidence suggests that MPNs may be underreported in US cancer registries [30]. It is unknown whether and to what extent MPN cases captured by SEER may differ from unreported cases.

Despite these limitations, our study provides new data on expected survival in Medicare enrollees with MPNs and supports new findings in a recent study of Swedish MPN patients that ET and PV do not carry as favorable a prognosis as once thought. These findings affirm a changing thought paradigm that recognizes all MPN subtypes as diseases that reduce life expectancy. Our findings may therefore have implications for the clinical management of MPN patients and underscore the need for improved therapies of all MPN subtypes. Further research is needed to not only assess MPN survival in a more generalized US population over a longer period of time, but to also formally examine potential patient and clinical factors that affect survival. Such information may further aid clinicians in the provision of optimal care for MPN patients and in the development of more effective therapies for all MPN subtypes.

## Supporting Information

Figure S1 Sample Attrition. ET = essential thrombocythemia, PV = polycythemia vera, MF = myelofibrosis, HMO = health maintenance organization, *MPN-NOS = 1,664 patients were identified in the study time period with a new diagnosis of MPN-NOS (myeloproliferative neoplasm-not otherwise specified) and excluded from this report.
(PDF)
