# Supplementary material for: Survival Patterns in United States (US) Medicare Enrollees with Non-CML Myeloproliferative Neoplasms (MPN)
Source: PLoS One. 2014 Mar 11;9(3):e90299. doi: 10.1371/journal.pone.0090299 (PMC3949729; doi:10.1371/journal.pone.0090299)
Supplement: Figure S1 — Sample Attrition. ET = essential thrombocythemia, PV = polycythemia vera, MF = myelofibrosis, HMO = health maintenance organization, *MPN-NOS = 1,664 patients were identified in the study time period with a new diagnosis of MPN-NOS (myeloproliferative neoplasm-not otherwise specified) and excluded from this report. (PDF) [file pone.0090299.s001.pdf]

Figure S-1. Sample Attrition

New MPN diagnosis between Jan 1, 2001 and Dec 31, 2007		
**ET**	**PV**	**MF**
2,421	3,432	1,040

↓

Has at least 1 month of Medicare enrollement during the study period (January 1, 2001 through December 31, 2007)		
2,389	3,376	1,022

↓

No HMO enrollment after first MPN diagnosis		
1,720	2,502	760

↓

Reason for Medicare eligibility is age or disability, not ESRD		
1,711	2,460	754

↓

1st MPN Diagnosis on/after 1st Medicare enrollment		
1,547	2,137	662

↓

Has ≥5 matched non-cancer controls (Final Sample)		
**ET**	**PV**	**MF**
1,217	1,625	522

ET = essential thrombocythemia, PV = polycythemia vera,
MF = myelofibrosis, HMO = health maintenance organization

*MPN-NOS = 1,664 patients were identified in the study time period with a new diagnosis of MPN-NOS (myeloproliferative neoplasm-not otherwise specified) and excluded from this report
